# Oncologic and obstetrical outcomes with fertility-sparing treatment of cervical cancer: a systematic review and meta-analysis

**DOI:** 10.18632/oncotarget.16233

**Published:** 2017-03-15

**Authors:** Qing Zhang, Wenhui Li, Margaux J. Kanis, Gonghua Qi, Minghao Li, Xingsheng Yang, Beihua Kong

**Affiliations:** ^1^ Department of Obstetrics and Gynecology, Qilu Hospital, Shandong University, Ji'nan, Shandong, P.R. China; ^2^ Gynecology Oncology Key Laboratory, Qilu Hospital, Shandong University, Ji'nan, Shandong, P.R. China; ^3^ Department of Obstetrics and Gynecology, Division of Gynecologic Oncology, Northwestern University Feinberg School of Medicine, Chicago, IL, USA; ^4^ Shandong University School of Medicine, Ji'nan, Shandong, P.R. China; ^5^ Department of Obstetrics and Gynecology, Peking Union Medical College Hospital, Chinese Academy of Medical Sciences & Peking Union Medical College, Beijing, China

**Keywords:** conization, radical trachelectomy, early cervical cancer, fertility-sparing treatment, live births

## Abstract

**OBJECTIVE:**

The objectives of this study were to evaluate the rates of recurrence, survival and pregnancy, and characterize pregnancy outcomes of early-stage cervical cancer(eCC) treated with fertility-sparing methods such as cervical conization (CON) and radical trachelectomy(RT) with or without pelvic lymphadenectomy.

**STUDY DESIGN:**

This was a meta-analysis of observational studies analyzed by a random-effects model and a meta-regression to assess heterogeneity.

**RESULTS:**

Sixty observational studies encompassing 2,854 patients were included; 17 of which evaluated CON and 43 RT. Three hundred and seventy-five patients were included in the CON group: 176(46.9%) stage IA1 and 167(44.5%) stage IB1. In the RT group, 2479 cases were included: 143(6.0%) stage IA1, 299(12.1%) stage IA2, 1987(79.9%) stage IB1. CON was performed in 347(92.5%) cases, resulting in a recurrence rate of 0.4%(95%CI: 0.0%-1.4%), a death rate of 0%(0%-0%), a pregnancy rate of 36.1%(26.4%-46.2%), a spontaneous abortion rate of 14.8%(9.3%-21.2%) and a preterm delivery rate of 6.8%(1.5%-15.5%). For the RT group, 2273(91.7%) underwent successful surgeries with a recurrence rate of 2.3%(1.3%-3.4%),a death rate of 0.7%(0.3%-1.1%), a pregnancy rate of 20.5%(16.8%-24.5%), a spontaneous abortion rate of 24.0%(18.8%-29.6%) and a preterm delivery rate of 26.6%(19.6%-34.2%). From a subgroup analysis, the recurrence rates for stage IA tumors treated with CON and RT were 0.4%(0.0%-1.9%) and 0.7%(0.0%-2.3%), respectively; and for stage IB were 0.6%(0.0%-2.7%) and 2.3%(0.9%-4.1%).

**CONCLUSION:**

Fertility-sparing treatment including CON or RT for eCC is feasible and carefully selected women can preserve fertility and achieve pregnancy resulting in live births. CON seems to result in better pregnancy outcomes than RT with similar rates of recurrence and mortality.

## INTRODUCTION

Cervical cancer is one of the most common gynecologic malignancy throughout the world. Standard treatment for cervical cancer includes a simple hysterectomy, a radical hysterectomy with pelvic lymphadenectomy or definitive chemoradiation, all of which result in the loss of childbearing ability. Recently, with the improvement of cervical cancer screening, and a trend towards childbearing at an older age, fertility-sparing surgery offers an attractive option for reproductive-aged women. As previously published, 43% of patients diagnosed with cervical cancer are under the age of 45 [1],and 20-28% are under the age of 40 [2, 3].

In women with early stage cervical cancer (eCC), it has been proposed that a trachelectomy (RT) or conization procedure (CON) are two options that allow for preservation of fertility. Dargent first published his experience with radical vaginal trachelectomies with a laparoscopic pelvic lymphadenectomy for young women with eCC in 1994 with excellent oncologic outcomes [4–8].To date, thousands of young patients have undergone trachelectomies resulting in a combined pregnancy rate of 24% and a recurrence and death rate of 4.2% and 2.9%, respectively [9]. Alternative approaches including abdominal, laparoscopic and robotic have been adopted.

Although research demonstrates that RT is a safe and feasible technique that can bring excellent clinical results similar to that of the standard surgical procedures for eCC, there are subsequent high rates of first and second trimester abortions and preterm births. In eCC, we know the rate of parametrial metastasis is low, supporting the trend towards performing even more conservative surgery such as a simple conization including a cold knife cone or loop electrical excision procedure (LEEP) with or without pelvic lymphadenectomy. To ascertain the efficacy of these therapies, we conducted a systematic review of observational studies to evaluate recurrence, mortality and pregnancy outcomes in the treatment of eCC in reproductive-age females, and performed a meta-analysis of their treatment effects.

## RESULTS

### Selection and quality analysis of the primary studies

**Figure 1:**
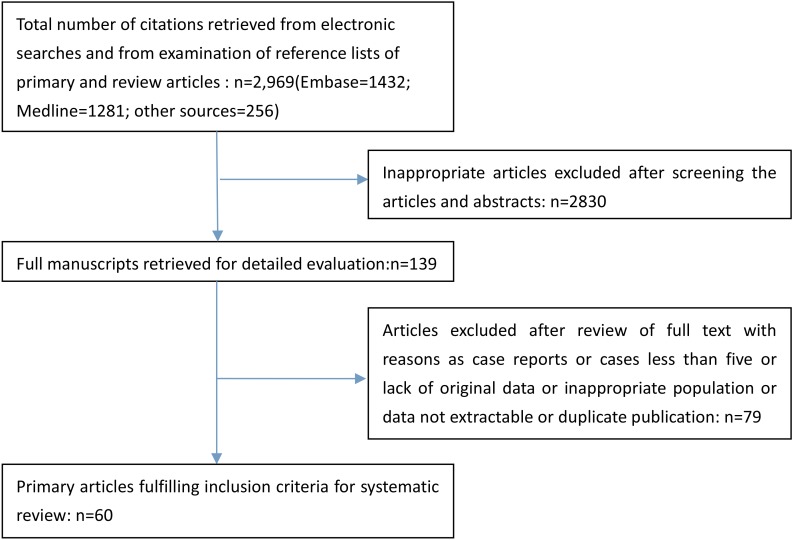
Flowchart of literature selection process

**Figure 2:**
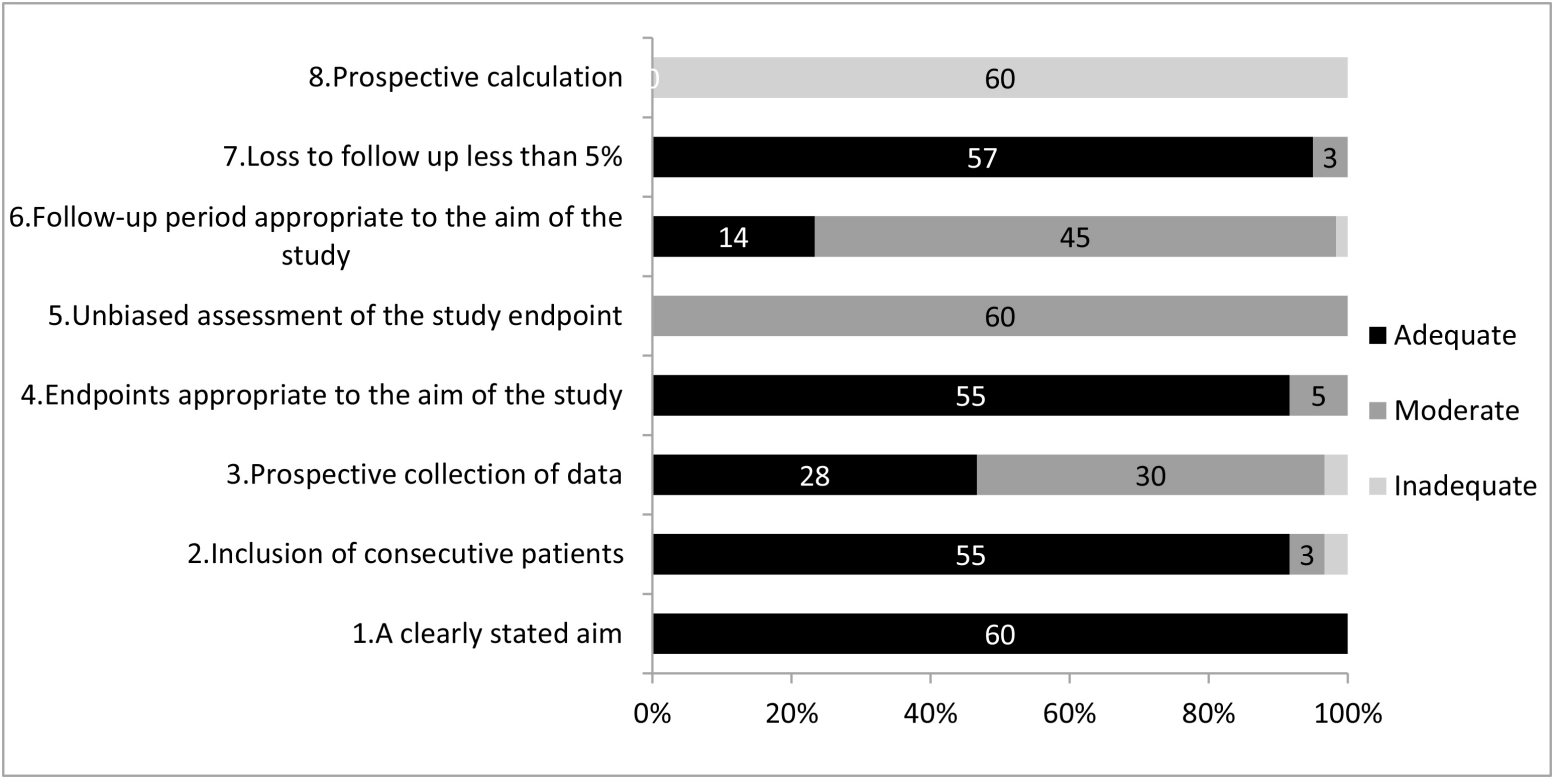
Quality assessment

We identified a total of 2969 articles and excluded 2830 as they did not meet criteria. The remaining 139 articles were reviewed and a further 79 were excluded (see Figure 1). The final analysis included 60 studies, totaling 2,854 patients. Seventeen studies focused on conization with 375 patients, and 43 studies reported on RT with 2479 women [2, 10–68]. The detailed information of the 60 studies is summarized in the Table 1. The methodologic index and qualities of all the studies according to the MINORS checklist are shown in Figure 2.

**Table 1:** Characteristic of the studies

NO.	Author,year		Stage					Intervention	Women Treated	Follow-up(Median, range inmonths)
		IA1	IA2	IB1	IB2		IIA			
CON01	Salihi R et al[10],2015(n=11)	0	0	10	1		0	conization	9	58
CON02	Ditto A et al[11], 2015(n=22)	0	6	16	0		0	conization	18	48.8
CON03	Fanfani F et al[12], 2014(n=23)	0	7	16	0		0	conization	23	40(32−125)
CON04	Min CC et al[13], 2014(n=21)	10	1	9	0		1	conization	21	52.6(6—114)
CON05	Andikyan V et al[14], 2014(n=10)	7	0	3	0		0	conization	9	17(1-83)
CON06	Biliatis I et al[15], 2012(n=35)	0	0	35	0		0	conization	33	56(13–132)
CON07	Maneo A et al[16], 2011(n=36)	0	0	36	0		0	conization	31	66(6–168)
CON08	Fagotti A et al[17], 2011(n=17)	0	4	13	0		0	conization	13	16(8-101)
CON09	Baalbergen A et al[18], 2011(n=22)	15	7	0	0		0	conization	20	79.9(10-131)
CON10	Yahata T et al[19], 2010(n=10)	10	0	0	0		0	conization	10	75(61-127)
CON11	Lee SJ et al[20], 2009(n=85)	85	0	0	0		0	conization	85	81.0(13-127)
CON12	Maneo A et al[21], 2008(n=21)	0	0	21	0		0	conization	16	69(10–124)
CON13	Bisseling KCHM et al[22], 2007(n=18)	16	2	0	0		0	conization	16	72
CON14	Landoni F et al[23], 2007(n=11)	0	3	8	0		0	conization	11	20(7–29)
CON15	Itsukaichi M et al[24], 2003(n=7)	7	0	0	0		0	conization	7	48(27.6–91.2)
CON16	Tseng CJ et al[25], 1997(n=12)	12	0	0	0		0	conization	12	80.4(60-111.6)
CON17	Morris M et al[26], 1993(n=14)	14	0	0	0		0	conization	13	26.5(1-170)
RT01	Hauerberg L et al[28], 2015(n=120)	9	8	103	0		0	RT	118	55.7(5.5–147)
RT02	Vieira MA et al[27], 2015(n=100)	6	25	69	0		0	RT	83	51(10–147)
RT03	Jeong-Yeol P et al[2], 2014(n=55)	0	2	53	0		0	RT	55	37(3-105)
RT04	Lanowska M et al[29], 2014(n=20)	0	0	15	4		1	RT	18	23.1(1-88)
RT05	Faber-Swensson AP et al[30], 2014(n=17)	0	4	13	0		0	RT	17	66(12–156)
RT06	Ma LK et al[31], 2014(n=46)	4	4	38	0		0	RT	46	39.5(1-77)
RT07	van Gent MD et al[32], 2014(n=28)	0	3	22	3		0	RT	28	47.3(6-122)
RT08	Park JY et al[33],2014(n=79)	0	4	72	2		1	RT	79	44(3-105)
RT09	Kucukmetin A et al[34], 2014(n=11)	0	0	11	0		0	RT	10	9(1-20)
RT09′	(n=16)	0	0	16	0		0	RT	16	43(8-110)
RT10	Capilna ME et al[35], 2014(n=26)	0	11	14	1		0	RT	23	20(4-43)
RT11	Lintner B et al[36], 2013(n=31)	0	0	17	14		0	RT	31	90(60-148)
RT12	Lu Q et al[37], 2013(n=25)	0	10	15	0		0	RT	25	66(1-82)
RT13	Nishio H et al[38], 2013(n=114)	9	12	93	0		0	RT	114	33
RT14	Ebisawa K et al[39], 2013(n=56)	0	4	52	0		0	RT	50	60(4-138)
RT15	Wethington SL et al[40], 2013(n=29)	0	0	29	0		0	RT	9	44(1-90)
RT16 RT16′	Cao DY et al[41], 2013(n=77) (n=73)	13 5	9 10	55 58	0 0		0 0	RT RT	71 55	24.5(6-91) 24.5(6-91)
RT17	Uzan C et al[42], 2013(n=28)	0	6	22	0		0	RT	27	59(3-132)
RT18	Li J et al[43], 2013(n=62)	0	0	62	0		0	RT	55	30.2 (2-108)
RT19	Testa R et al[44], 2013(n=25)	0	6	19	0		0	RT	24	29.6
RT20	Muraji M et al[45], 2012(n=8)	0	0	8	0		0	RT	7	20(2-32)
RT20′	(n=15)	2	2	11	0		0	RT	12	37.5(30-46)
RT21	Kim CH et al[46], 2012(n=105)	14	12	79	0		0	RT	77	/
RT22	Persson J et al[47], 2012(n=12)	4	2	6	0		0	RT	10	76(48–115)
RT22′	(n=13)	4	5	4	0		0	RT	12	24(6-54)
RT23	Raju SK et al[48], 2012(n=51)	0	2	49	0		0	RT	47	96(12-120)
RT24	Nick AM et al[49], 2012(n=37)	5	11	21	0		0	RT	32	17.0(0.30–64.9)
RT25	Wethington SL et al[50], 2012(n=101)	3	8	88	1		1	RT	70	32(1-124)
RT26	Saso S et al[51], 2012(n=30)	0	2	25	2		1	RT	28	24(7-113)
RT27	Plante M et al[52], 2011(n=125)	7	29	85	2		2	RT	119	95(4-225)
RT28	Li J et al[53], 2011(n=59)	16	7	36	0		0	RT	59	22.8(1-78)
RT29	Marchiole P et al[54], 2011(n=7)	0	0	2	3		2	RT	7	22(5–49)
RT30	Speiser D et al[55], 2011(n=212)	34	47	131	0		0	RT	212	>12
RT31	Yao T et al[56], 2010(n=10)	0	5	5	0		0	RT	10	(4-68)
RT32	Kim JH et al[57], 2010(n=27)	0	0	26	0		1	RT	27	31(1-58)
RT33	Shepherd JH et al[58], 2009(n=142)	0	2	139	1		0	RT	142	57
RT34	Olawaiye A et al[59], 2009(n=10)	1	3	5	0		1	RT	10	28(1-66)
RT35	Cibula D et al[60], 2009(n=24)	0	2	22	0		0	RT	17	21.2
RT36	Nishio H et al[61], 2009(n=61)	4	8	49	0		0	RT	61	27(1-67)
RT37	Sonoda Y et al[62], 2008(n=40)	0	0	40	0		0	RT	40	44(3-201)
RT38	Milliken DA et al[63], 2008(n=158)	0	4	152	0		2	RT	138	/
RT39	Pareja FR et al[64], 2008(n=15)	0	3	12	0		0	RT	15	32(5-32)
RT40	Shepherd JH et al[65], 2006(n=123)	0	2	121	0		0	RT	112	45(1–120)
RT41	Chen Y et al[66], 2006(n=16)	3	7	6	0		0	RT	16	28.2(8-50)
RT42	Ungar L et al[67], 2005(n=30)	0	10	15	5		0	RT	29	32(14-75)
RT43	Schlaerth JB et al[68], 2003(n=10)	0	8	2	0		0	RT	10	10persons≥24, 2≥60

### Patient characteristics

The median age of patients included ranged from 27 to 39 years old. The length of follow-up was 9-95 months. Based on NCCN guidelines, we considered an appropriate follow-up to be at least 5 years, although only 14 of the 60 published results that included this length of surveillance. In 37 papers, patients underwent the diagnostic imaging such as MRI, CT or PET to exclude distant disease and lymphatic metastasis. The qualities of all the studies according to the MINORS checklist have been shown in Figure 2.

### Prognosis and pregnancy outcome of fertility-sparing treatment by conization

The 17 CON studies identified 375 cases including 176(46.93%) women with stage IA1, 30(8.00%) with stage IA2, 167(44.53%) with stage IB1, 1(0.27%) with stage IB2, 1(0.27%) with stage IIA. 347 (92.5%) successfully underwent the fertility-sparing treatment. The median follow-up in this group ranged from 16 to 81 months and 82.4%(14/37) were followed for more than 2 years.

Among the 347 who underwent conization, 4 relapsed with a pooled proportion and its 95%CIs from the forest plot to be 0.4%(95%CI: 0.0%-1.4%) (sFigure 1). The value for I^2^ test was 0% indicating no significant heterogeneity between the studies(*P* > 0.05). Disease-specific mortality after conization was 0%(0%-0%) with no obvious heterogeneity(I^2^ = 0%, P > 0.05).

Meta-analysis of the 37 studies reporting pregnancy results found that 113 young women achieved at least 1 pregnancy after conization resulting in a pregnancy rate of 36.1%(26.4%-46.2%) with high heterogeneity(I^2^ = 71.0%, *P* < 0.05). Among all patients who conceived, the spontaneous abortion rate was 14.8%(9.3%-21.2%) with moderate heterogeneity(I^2^ = 39.8%, *P* > 0.05). Similarly, we obtained a preterm delivery rate of 6.8%(1.5%-15.5%) with moderate heterogeneity(I^2^ = 40.3%, *P* > 0.05).

### Prognosis and pregnancy outcome of fertility-sparing treatment by RT

We enrolled 43 articles about RT(RT group) with 2479 cases including 143(6.0%)women with stage IA1, 299(12.1%) with stage IA2, 1987(79.9%) with stage IB1. 2273 of 2479 patients(91.7%) received the fertility-conserving treatment. In these 43 studies, patients were followed up over time with the average ranging from 9 to 96 months and 81.4%(35/47) was more than 2 years.

**Figure 3:**
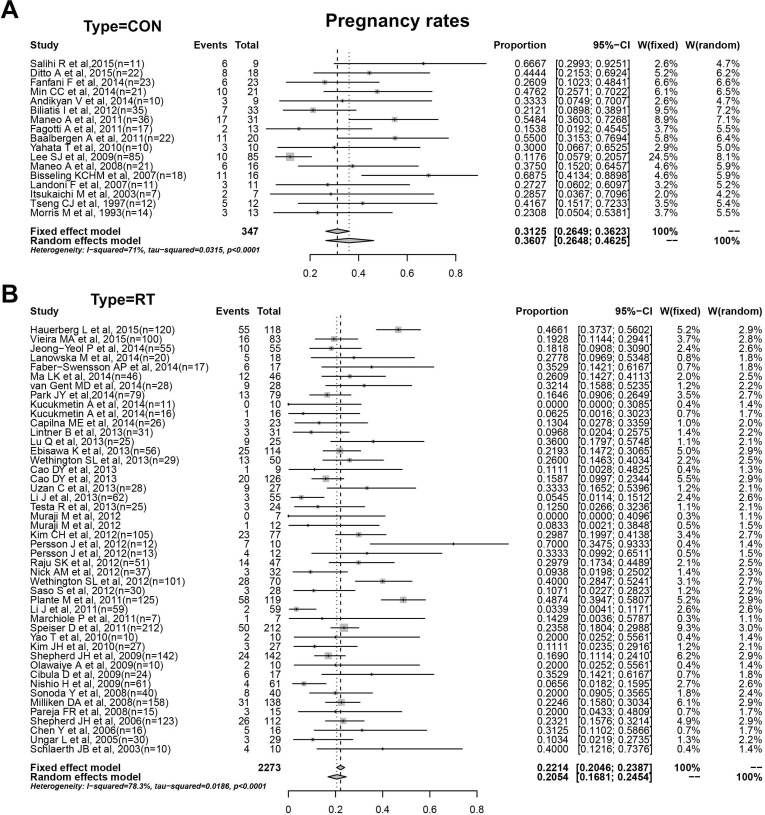
**A**. Pregnancy rates of conization. **B**. Pregnancy rates of RT.

**Figure 4:**
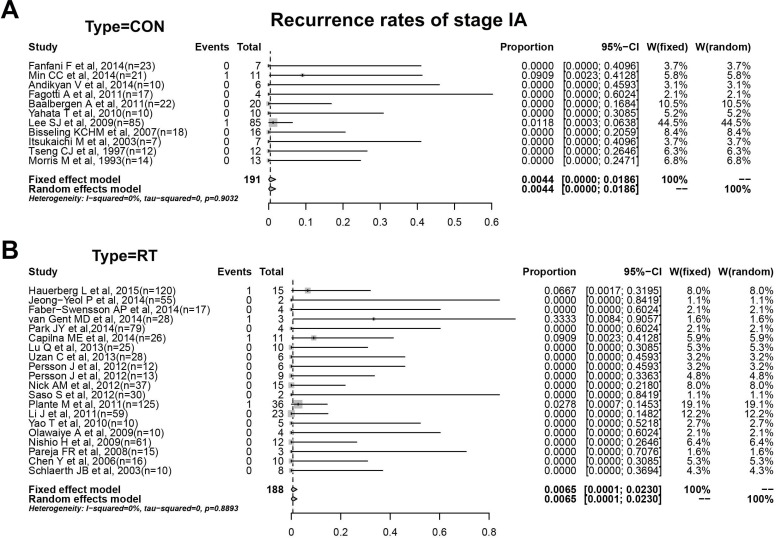
**A**. Recurrence rates of conization with stage IA. **B**. Recurrence rates of RT with stage IA.

**Figure 5:**
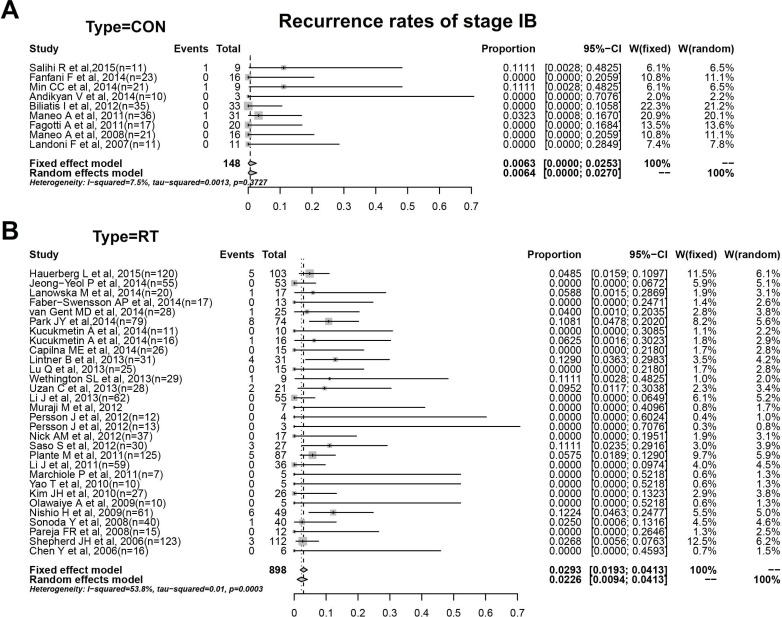
**A**. Recurrence rates of conization with stage IB. **B**. Recurrence rates of RT with stage IB.

Meta-analysis of the 43 studies with fertility-conservative treatment by RT amounted to the pooled estimates of the corresponding rates and 95%CIs from the forest plots shown in Figure 3B-5B and sFigure 1-04. The relapse and dead cases were reported in all articles and concluded their respective pooled occurrence rates in the forest plots to be 2.3%(1.3%-3.4%) with obvious heterogeneity and 0.7%(0.3%-1.1%) with minor heterogeneity. In these 43 studies indicated, we found 531 women after conservative treatment conceive at least once, which amounted to a pooled pregnancy rate of 20.5%(16.8%-24.5%) from the forest plot. The value of I^2^ test was 78.3%, indicating the existence of heterogeneity. There were 805 pregnancies recorded during follow-up, from which several cases had an abortion in first- or second-trimester, amounting to a pooled abortion rate of 24.0%(18.8%-29.6%) by meta-analysis with obvious heterogeneity between studies(I^2^ = 55.5%, *P* < 0.05). As well, the preterm delivery rate was computed in the forest plot to be 26.6%(19.6%-34.2%) finally, with I^2^ = 75.6%(*P* < 0.05). The results of the two groups were concluded in Table 2.

**Table 2:** Results of the proportions from forest plots

Proportions	Number of studies	Test for Heterogeneity(I^2^)	Test for Heterogeneity: (p value of Q test)	Fixed effect model (effect size,95%CI)	Random effects model (effect size,95%CI)	*p*
recurrence rate of appendix involved	63	49.7%	< 0.0001	0.0231 [0.0176; 0.0294]	0.0173 [0.0102; 0.0263]	0.0116
In CON	17	0.0%	0.8427	0.0042 [0.0002; 0.0137]	0.0042 [0.0002; 0.0137]	
In RT	46	55.2%	< 0.0001	0.0277 [0.0211; 0.0351]	0.0225 [0.0133; 0.0341]	
death rate of appendix involved	62	0.0%	0.5428	0.0055 [0.0030; 0.0089]	0.0055 [0.0030; 0.0089]	0.0188
In CON	17	0.0%	0.9994	0.0003 [0.0000; 0.0047]	0.0003 [0.0000; 0.0047]	
In RT	45	11.9%	0.2485	0.0071 [0.0039; 0.0111]	0.0065 [0.0032; 0.0108]	
pregnancy rate of appendix involved	63	77.5%	< 0.0001	0.2329 [0.2169; 0.2492]	0.2381 [0.2018; 0.2765]	0.0011
In CON	17	71.0%	< 0.0001	0.3125 [0.2649; 0.3623]	0.3607 [0.2648; 0.4625]	
In RT	46	78.3%	< 0.0001	0.2214 [0.2046; 0.2387]	0.2054 [0.1681; 0.2454]	
abortion rate of appendix involved	60	55.6%	< 0.0001	0.2529 [0.2255; 0.2814]	0.2056 [0.1612; 0.2539]	0.0182
In CON	17	39.8%	0.0511	0.1475 [0.0934; 0.2116]	0.1196 [0.0558; 0.2032]	
In RT	44	55.5%	< 0.0001	0.2732 [0.2427; 0.3048]	0.2395 [0.1875; 0.2957]	
preterm delivery rate of appendix involved	57	75.5%	< 0.0001	0.2851 [0.2559; 0.3152]	0.2134 [0.1537; 0.2801]	0.0020
In CON	17	40.3%	0.0653	0.0778 [0.0320; 0.1413]	0.0679 [0.0148; 0.1553]	
In RT	46	75.6%	< 0.0001	0.3145 [0.2827; 0.3471]	0.2660 [0.1961; 0.3423]	
recurrence rate of stage IA	31	0.0%	0.9748	0.0054 [0.0005; 0.0152]	0.0054 [0.0005; 0.0152]	0.7757
In CON	11	0.0%	0.9032	0.0044 [0.0000; 0.0186]	0.0044 [0.0000; 0.0186]	
In RT	20	0.0%	0.8893	0.0065 [0.0001; 0.0230]	0.0065 [0.0001; 0.0230]	
recurrence rate of stage IB	39	49.8%	0.0003	0.0250 [0.0164; 0.0354]	0.0190 [0.0082; 0.0341]	0.2616
In CON	9	7.5%	0.3727	0.0063 [0.0000; 0.0253]	0.0064 [0.0000; 0.0270]	
In RT	30	53.8%	0.0003	0.0293 [0.0193; 0.0413]	0.0226 [0.0094; 0.0413]	

### Prognosis of patients with stage IA or IB after conization and RT

Eleven studies including 191 women were enrolled in the analysis of prognosis result by conization with stage IA. As shown in Figure 4A, we obtained pooled proportion and CIs by mete-analysis from the forest plots. Two cases of recurrence amounted to a recurrent rate of 0.4%(0.0%-1.9%) with no obvious heterogeneity(I^2^ = 0, *P* > 0.05). Meanwhile, we included 20 studies with 188 patients of stage IA enrolled in the analysis of prognosis by RT, which amounted to a recurrent rate in RT group of 0.7%(0.0%-2.3%) with no obvious heterogeneity(I^2^ = 0, *P* > 0.05).

For stage IB, 148 patients(99% IB1) in CON group were included for assessment of prognosis by conization. In Figure 5A, 3 cases of recurrence amounted to a recurrence rate of 0.6%(0.0%-2.7%) with minor heterogeneity(I^2^ = 7.5%, *P* > 0.05). And we enrolled 30 studies with 898 patients of stage IB(97% IB1) in the analysis of prognosis by RT(Figure 5B). The recurrence rate in RT group obtained from forest plot were 2.3%(0.9%-4.1%) with obvious heterogeneity(I^2^ = 53.8%, *P* < 0.05). The results by conization or RT in different stages were summarized in Table 2.

## DISCUSSION

Fertility preservation and quality of life are becoming increasingly important concerns of young women with cervical cancer and less invasive surgical procedures have become a potential solution. The results of this review demonstrate that conization has similar oncologic outcomes to RT for eCC patients especially those before stage IB1 to preserve fertility. Furthermore, obstetrical outcomes are very encouraging, underscoring the role of conization in these patients.

What's more, the minimally invasive surgery have confirmed the advantages of a reduced length of hospital stay, less blood loss, lower analgesic requirements during the post operative period, a decreased rate of complications, and an early recovery of physiological functions [69, 70]. Numerous researches have proved that RT can obtain a similar death rate with radical hysterectomy in low-risk eCC to be a safe alternative for fertility conserving. Some surveys shown that recurrence rate of Dargent's operation was reported to be 4.2%-4.7% and mortality rate between 2.8% and 3.0% [71, 72]. In 2013, Plante M reviewed articles and generalized the recurrence rate of ART and LRT to be 4% and 7% respectively. In our review, we do a meta-analysis and it shows a low recurrence rate of 2.3% (1.3%-3.4%) and mortality of 0.7%(0.3%-1.1%) in RT group, which are close to the aforesaid statistics. However, although RT is quite effective from the oncologic point of view, the execution of this procedure to maintain pregnancy competence for young eCC patients responds a bit undesirable conception outcome with a high risk of first- and second-trimester miscarriage and preterm delivery [68, 73–75]. There have been more than 300 pregnancies reported after RT in the literature, with a live-birth rate of 68% [76]. But literature by Rob et al showed that pregnancy loss or delivery before 32 weeks was as high as 44% for VRT and 38% for ART [71], and Pareja et al. in 2015 concluded the global pregnancy rates of 16.2% in ART and 24% in VRT [77]. The data seem to range broadly because it may be calculated in different ways or influenced by the number of women attempting to conceive among patients after conservative procedure. With respect to our research, we did a meta-analysis and the aggregated rates of RT in miscarriage and preterm labor are 24.0%(18.8%-29.6%) and 26.6%(19.6%-34.2%) separately with a pooled pregnancy rate of 20.5%(16.8%-24.5%) in all fertility-conserved ones.

Compared with results of RT, conization is more likely to be encouraging in the aspects of pregnancy possibility and pregnancy ending due to less parametrial excision and mild damage to pelvic floor, which might benefit decreasing rates of pregnancy loss. But information about conization security and pregnancy outcome is still scant. For many retrospective researches have revealed that in small-volume, low-risk, early-stage cervical cancer (defined as measuring < 2cm with < 50% stromal invasion), the probability of parametrial extension is very low and even less than 1% concluded from a 1000 patient retrospective review analysis, which testified indirectly the security of conization [78]. In addition, previous studies have shown that patients who undergo a RT have no residual disease accounts for approximately 60% in their surgical specimen, suggesting that perhaps those patients could have been treated with less radical surgery [79]. Plante also pointed that albeit no sufficient and mature data were available so far, large conization(in low-risk patients) seem to support oncologic safety of radical trachelectomy, improving reproductive outcomes. Ditto et al. evaluated the safety, feasibility and effectiveness of conization plus laparoscopic pelvic lymphadenectomy in eCC women of stage IA2-IB1 and concluded 5-year disease-free and overall survival rates of 85.9% and 93.7%, respectively with a 44% spontaneous pregnancy rate [11].

In addition, Lindsay et al reported encouraging oncologic (recurrence rate, 5%) and reproductive(live birth rate, 35%) outcomes undergoing large loop excision of the transformation zone and laparoscopic pelvic lymph node dissection [80]. In our meta-analysis, the 17 essays referred have low recurrence rates ranging from 0 to 11%, summarized to be 0.4%(0.0%-1.4%), with corresponding death rate of 0%(0%-0%), which shows quite ideal results close to those of RT procedure. And moreover, to get rid of the influence of stages on prognosis, we did subgroups with stage IA and IB(mainly IB1) which also showed great outcome in CON group by recurrent rates compared with RT. But what we want to emphasize here is that conization management seem to receive much better results than RT in pregnancy incidence 36.1%(26.4%-46.2%), and the miscarriage and premature birth rate of conization are much lower for 14.8%(9.3%-21.2%) and 6.8%(1.5%-15.5%) respectively among all reproductive-conserved patients.

However, as we reviewed large amounts of articles, young patients with eCC treated by RT or even conization as fertility-sparing treatment must be screened out with strict inclusion criteria probably referring to the age of patients, stages, histological subtype, tumor volume, depth of invasion, lymphatic spread, or parametrial involvement. Most oncologists maintain that tumors measuring < 2cm are one of the key requirements, particularly for conization, to guarantee removal of all tumor. Some researchers believed patients with tumors larger than 2 cm had a higher recurrent rate than those smaller than 2 cm, and when LVSI are positive, wider resection are needed; for patients with tumors larger than 2 cm, ART or neoadjuvant chemotherapy are more recommended for conservative treatment to gain a similar recurrent rate. But on the other side, some studies showed unclear difference with prognosis under different tumor volume and LVSI state. Owing to the limited articles and information we referred and absence of randomized trials comparing different therapeutic conservative strategies, we didn't analyze the different effect of stages, tumor volume, or other factors such as chemotherapy and in our study. But there are still some patients with larger tumors showing a favorable outcome after conservative surgeries and the “one-size-fits-all” concept is gradually to be challenged. This will be likely to be completed more broadly as the effectiveness of neoadjuvant chemotherapy are proved and applied one day. We included 20 papers referred to more than 300 patients with tumors larger than 2cm which couldn't be seperated for detailed discussion. In addition, application of chemotherapy after surgery was mentioned in several articles, primarily for patients after conization. 8 articles referred to patients receiving neoadjuvant chemotherapy were included in CON group which was believed to ensure the safety of conservative treatment and all studies showed ideal outcome according to the limited patients. But restricted by limited information, it couldn't be talked by subgroups. For limited excision, some surgeons hold the opinion that patients with increased risk of recurrence need to receive chemotherapy after conization, such as positive margin, tumor measuring > 2cm, deep stromal infiltration. And researchers [13] suggest no effect of chemotherapy on ovarian function in short follow-up.

However, the recurrence rates during follow-up are concerning and many factors relevant have not been discussed in this review to explain it further. And our study are carried on by a meta-analysis based on respective rates rather than RCTs which brings confounding effects and weakens the reliability and we only analyze patients without distinguishing tumors larger or smaller than 2 cm, adjuvant therapy or not, histological types for detailed discussion. Despite numerous studies confirming the oncologic and obstetrical validity of conization, and similarly, RT, additional prospective cohort studies are required.

## COMMENT

In our review, we found that both CON and RT with or without lymphadenectomy are encouraging as a fertility-sparing treatment for eCC, especially in stage IA-IB1, according to the low relapse rates of conization and RT, and an additional encouraging proportion of women managed to achieve pregnancy. For patients with stage IA, conization seems much suitable for lower abortion rate and preterm delivery rate, resulted from the limited and minor injury to the cervical and parametrium, and great oncologic outcome. For stage IB, particularly IB1, patients should be evaluated comprehensively before conservative treatment and conization with pelvic lymphadenectomy may be a suitable option.

## MATERIALS AND METHODS

### Search strategy and selection criteria

The study population in this review included women with eCC (predominantly International Federation of Gynecology and Obstetrics stage I) who desired to maintain fertility. Patients underwent fertility-sparing therapies including conization or RT with or without pelvic lymphadenectomy. The primary outcome measures were disease recurrence, mortality and pregnancy outcomes. Electronic searches using Medline, the Cochrane Library and Embase were performed for studies published in English between 1993 and September 2015. The terms used in the search were “cervical cancer”, “radical trachelectomy”, “conization”, “fertility sparing”, “oncological and obstetrical outcome”. Reference lists of all articles identified by our searches were reviewed to identify potential missing studies or unpublished data.

Articles were selected if the patients had eCC defined as stage IA-IB according to FIGO 2009 staging system, documented pathology review, underwent conization or RT for conservative therapy, and the results described the recurrence, mortality and pregnancy outcomes. Patients in studies were aged under 45 years old without evidence of infertility and other malignancies. We excluded case reports, review articles, series updates and series with less than five patients. All articles were checked independently by 2 reviewers. The references for retrieved articles together with the proceedings of relevant conferences were hand-searched to identify other potentially eligible publications that met the predefined selection criteria for inclusion in the analysis and missed by the initial search. For overlap or duplicate cases, the most recent or the most comprehensive publication was used. Disagreements were resolved by consensus or arbitration by a third reviewer. An additional two reviewers were used for the quality assessment. The Methodological Index for Non-Randomized Studies(MINORS) was used to evaluate the quality of the included studies [81].

### Data extraction and analysis

In this review, pregnancy rate was defined as the number of women who conceived successfully divided by the number of total of women who retained their fertility during follow-up. We counted the number of patients with recurrent disease and dead women to assess the oncologic outcomes of the two conservative surgeries. We calculated spontaneous abortion and premature birth rates for further description of pregnancy outcome.

Data on relapse, mortality, pregnancy, abortion, preterm delivery were extracted from each identified research, and we computed the log of the ratio and its corresponding standard error for each research. We calculated the random-effects summary estimates by meta-analysis using inverse-variance weighting. Forest plots were formed for each outcome, presenting individual study proportion with 95% confidence intervals (95%CIs), as well as the overall pooled estimates showing the corresponding ratio and 95%CIs. Heterogeneity of the treatment effects was also assessed by I^2^ analyzed graphically with forest plots. Statistical analysis was performed with R software(America).

To control for the effect of stage on prognosis, we performed a subgroup meta-analysis by stratifying outcomes by stage (IA and IB).

## SUPPLEMENTARY MATERIALS FIGURE


