# Supplementary material for: Oncologic and obstetrical outcomes with fertility-sparing treatment of cervical cancer: a systematic review and meta-analysis
Source: Oncotarget. 2017 Mar 15;8(28):46580–92. doi: 10.18632/oncotarget.16233 (PMC5542294; doi:10.18632/oncotarget.16233)
Supplement: Supplementary file 1 [file oncotarget-08-46580-s001.pdf]

Oncologic and obstetrical outcomes with fertility-sparing treatment of cervical cancer: a systematic review and meta-analysis

Supplementary Material

Study	Events	Total	Proportion	95%-CI	W(fixed)	W(random)
type = CON						
Salihi R et al,2015(n=11)	1	9	0.1111	[0.0028; 0.4825]	0.4%	0.7%
Ditto A et al, 2015(n=22)	0	18	0.0000	[0.0000; 0.1853]	0.7%	1.2%
Fanfani F et al, 2014(n=23)	0	23	0.0000	[0.0000; 0.1482]	0.9%	1.4%
Min CC et al, 2014(n=21)	1	21	0.0476	[0.0012; 0.2382]	0.9%	1.4%
Andikyan V et al, 2014(n=10)	0	9	0.0000	[0.0000; 0.3363]	0.4%	0.7%
Biliatis I et al, 2012(n=35)	0	33	0.0000	[0.0000; 0.1058]	1.3%	1.8%
Maneo A et al, 2011(n=36)	1	31	0.0323	[0.0008; 0.1670]	1.3%	1.7%
Fagotti A et al, 2011(n=17)	0	13	0.0000	[0.0000; 0.2471]	0.5%	1.0%
Baalbergen A et al, 2011(n=22)	0	20	0.0000	[0.0000; 0.1684]	0.8%	1.3%
Yahata T et al, 2010(n=10)	0	10	0.0000	[0.0000; 0.3085]	0.4%	0.8%
Lee SJ et al, 2009(n=85)	1	85	0.0118	[0.0003; 0.0638]	3.5%	2.6%
Maneo A et al, 2008(n=21)	0	16	0.0000	[0.0000; 0.2059]	0.7%	1.1%
Bisseling KCHM et al, 2007(n=18)	0	16	0.0000	[0.0000; 0.2059]	0.7%	1.1%
Landoni F et al, 2007(n=11)	0	11	0.0000	[0.0000; 0.2849]	0.4%	0.9%
Itsukaichi M et al, 2003(n=7)	0	7	0.0000	[0.0000; 0.4096]	0.3%	0.6%
Tseng CJ et al, 1997(n=12)	0	12	0.0000	[0.0000; 0.2646]	0.5%	0.9%
Morris M et al, 1993(n=14)	0	13	0.0000	[0.0000; 0.2471]	0.5%	1.0%
Fixed effect model		347	0.0042	[0.0002; 0.0137]	14.1%	--
Random effects model			0.0042	[0.0002; 0.0137]	--	20.2%
Heterogeneity: I-squared=0%, tau-squared=0, p=0.8427						
type = RT						
Hauerberg L et al, 2015(n=120)	6	118	0.0508	[0.0189; 0.1074]	4.8%	2.9%
Vieira MA et al, 2015(n=100)	1	83	0.0120	[0.0003; 0.0653]	3.4%	2.6%
Jeong-Yeol P et al, 2014(n=55)	0	55	0.0000	[0.0000; 0.0649]	2.2%	2.3%
Lanowska M et al, 2014(n=20)	1	18	0.0556	[0.0014; 0.2729]	0.7%	1.2%
Faber-Swensson AP et al, 2014(n=17)	0	17	0.0000	[0.0000; 0.1951]	0.7%	1.2%
Ma LK et al, 2014(n=46)	4	46	0.0870	[0.0242; 0.2079]	1.9%	2.1%
van Gent MD et al, 2014(n=28)	2	28	0.0714	[0.0088; 0.2350]	1.1%	1.6%
Park JY et al,2014(n=79)	9	79	0.1139	[0.0534; 0.2053]	3.2%	2.6%
Kucukmetin A et al, 2014(n=11)	0	10	0.0000	[0.0000; 0.3085]	0.4%	0.8%
Kucukmetin A et al, 2014(n=16)	1	16	0.0625	[0.0016; 0.3023]	0.7%	1.1%
Capilna ME et al, 2014(n=26)	1	26	0.0385	[0.0010; 0.1964]	1.1%	1.5%
Lintner B et al, 2013(n=31)	4	31	0.1290	[0.0363; 0.2983]	1.3%	1.7%
Lu Q et al, 2013(n=25)	0	25	0.0000	[0.0000; 0.1372]	1.0%	1.5%
Ebisawa K et al, 2013(n=56)	1	50	0.0200	[0.0005; 0.1065]	2.0%	2.2%
Wethington SL et al, 2013(n=29)	1	9	0.1111	[0.0028; 0.4825]	0.4%	0.7%
Cao DY et al, 2013	7	71	0.0986	[0.0406; 0.1926]	2.9%	2.5%
Cao DY et al, 2013	0	55	0.0000	[0.0000; 0.0649]	2.2%	2.3%
Uzan C et al, 2013(n=28)	2	27	0.0741	[0.0091; 0.2429]	1.1%	1.6%
Li J et al, 2013(n=62)	0	55	0.0000	[0.0000; 0.0649]	2.2%	2.3%
Testa R et al, 2013(n=25)	0	24	0.0000	[0.0000; 0.1425]	1.0%	1.5%
Muraji M et al, 2012	0	7	0.0000	[0.0000; 0.4096]	0.3%	0.6%
Muraji M et al, 2012	0	12	0.0000	[0.0000; 0.2646]	0.5%	0.9%
Kim CH et al, 2012(n=105)	0	23	0.0000	[0.0000; 0.1482]	0.9%	1.4%
Persson J et al, 2012(n=12)	0	10	0.0000	[0.0000; 0.3085]	0.4%	0.8%
Persson J et al, 2012(n=13)	0	12	0.0000	[0.0000; 0.2646]	0.5%	0.9%
Raju SK et al, 2012(n=51)	1	47	0.0213	[0.0005; 0.1129]	1.9%	2.1%
Nick AM et al, 2012(n=37)	0	32	0.0000	[0.0000; 0.1089]	1.3%	1.7%
Wethington SL et al, 2012(n=101)	4	70	0.0571	[0.0158; 0.1399]	2.9%	2.5%
Saso S et al, 2012(n=30)	3	28	0.1071	[0.0227; 0.2823]	1.1%	1.6%
Plante M et al, 2011(n=125)	6	119	0.0504	[0.0187; 0.1065]	4.8%	2.9%
Li J et al, 2011(n=59)	0	59	0.0000	[0.0000; 0.0606]	2.4%	2.3%
Marchiole P et al, 2011(n=7)	0	7	0.0000	[0.0000; 0.4096]	0.3%	0.6%
Speiser D et al, 2011(n=212)	8	212	0.0377	[0.0164; 0.0730]	8.6%	3.3%
Yao T et al, 2010(n=10)	0	10	0.0000	[0.0000; 0.3085]	0.4%	0.8%
Kim JH et al, 2010(n=27)	1	27	0.0370	[0.0009; 0.1897]	1.1%	1.6%
Shepherd JH et al, 2009(n=142)	4	142	0.0282	[0.0077; 0.0706]	5.8%	3.0%
Olawaiye A et al, 2009(n=10)	0	10	0.0000	[0.0000; 0.3085]	0.4%	0.8%
Cibula D et al, 2009(n=24)	1	17	0.0588	[0.0015; 0.2869]	0.7%	1.2%
Nishio H et al, 2009(n=61)	6	61	0.0984	[0.0370; 0.2019]	2.5%	2.4%
Sonoda Y et al, 2008(n=40)	1	40	0.0250	[0.0006; 0.1316]	1.6%	2.0%
Milliken DA et al, 2008(n=158)	4	138	0.0290	[0.0080; 0.0726]	5.6%	3.0%
Pareja FR et al, 2008(n=15)	0	15	0.0000	[0.0000; 0.2180]	0.6%	1.1%
Shepherd JH et al, 2006(n=123)	3	112	0.0268	[0.0056; 0.0763]	4.6%	2.9%
Chen Y et al, 2006(n=16)	0	16	0.0000	[0.0000; 0.2059]	0.7%	1.1%
Ungar L et al, 2005(n=30)	0	29	0.0000	[0.0000; 0.1194]	1.2%	1.6%
Schlaerth JB et al, 2003(n=10)	0	10	0.0000	[0.0000; 0.3085]	0.4%	0.8%
Fixed effect model		2108	0.0277	[0.0211; 0.0351]	85.9%	--
Random effects model			0.0225	[0.0133; 0.0341]	--	79.8%
Heterogeneity: I-squared=55.2%, tau-squared=0.0069, p<0.0001						
**Fixed effect model**		**2455**	**0.0231**	**[0.0176; 0.0294]**	**100%**	**--**
**Random effects model**			**0.0173**	**[0.0102; 0.0263]**	**--**	**100%**
Heterogeneity: I-squared=49.7%, tau-squared=0.0065, p<0.0001						

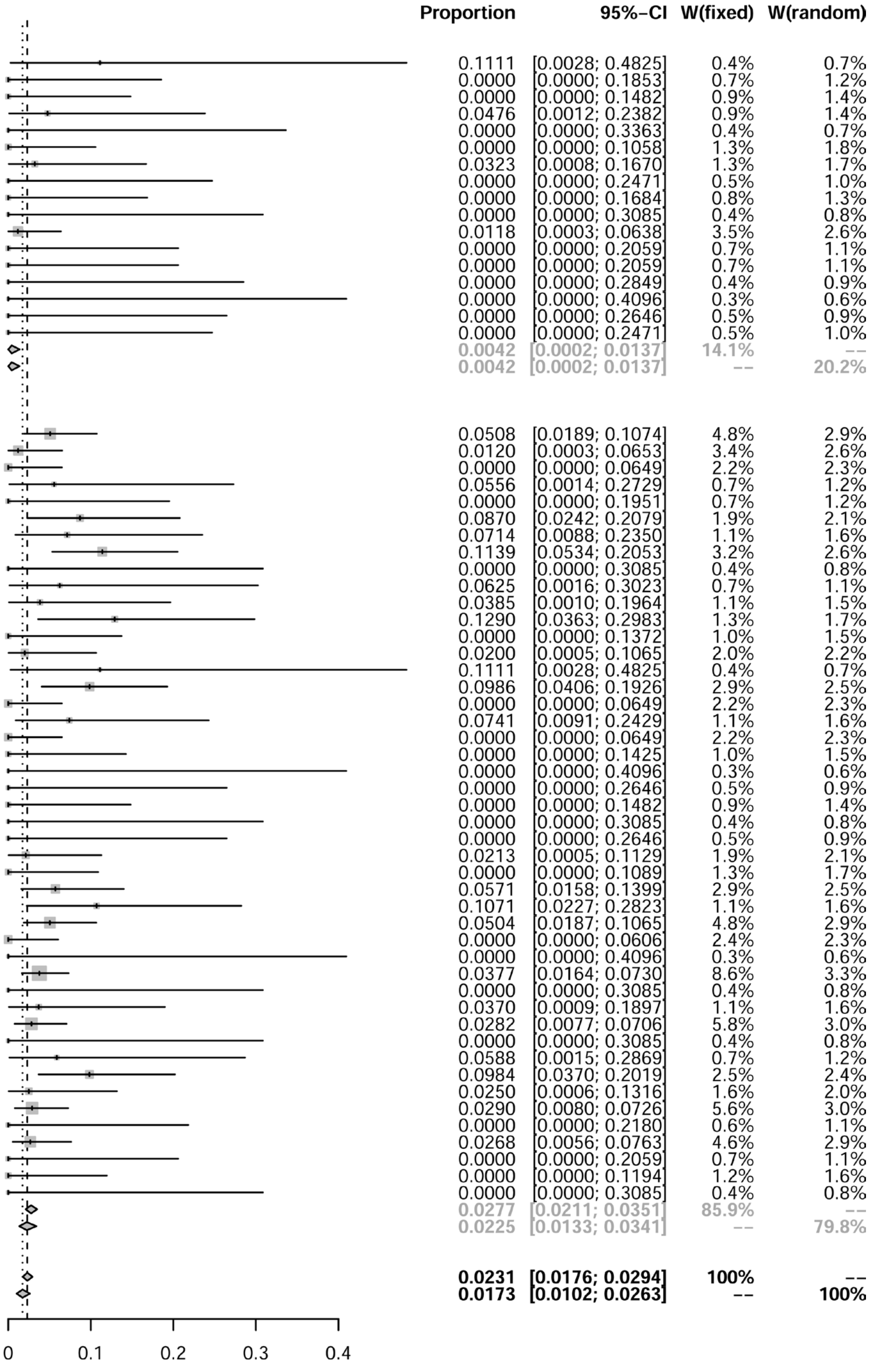

Supplementary Figure 1: Recurrence rates of conization and RT.

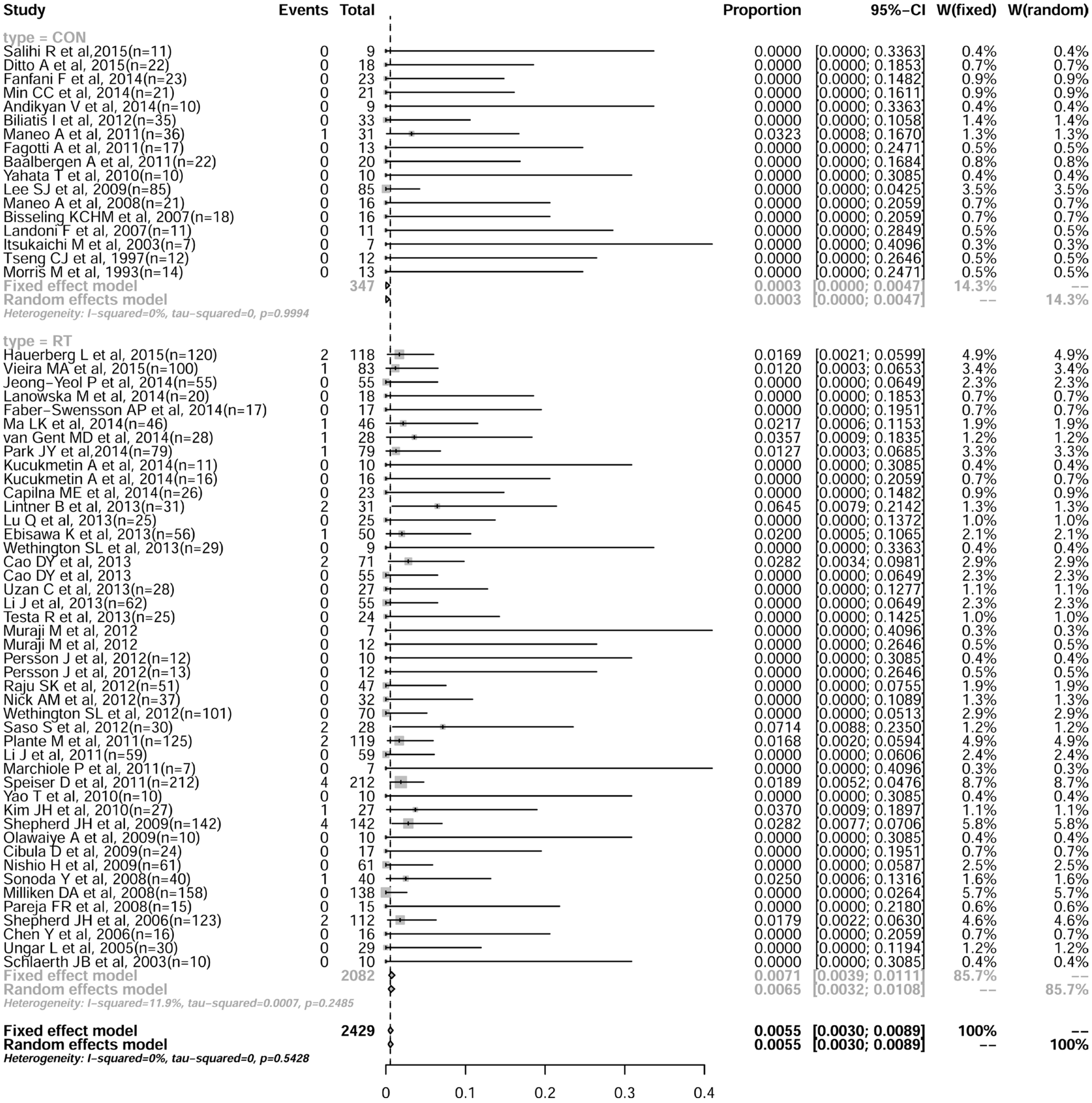

Supplementary Figure 2 Death rates of conization and RT.

Study	Events	Total	Proportion	95%-CI	W(fixed)	W(random)
**type = CON**						
Salihi R et al,2015(n=11)	4	11	0.3636	[0.1093; 0.6921]	1.2%	2.0%
Ditto A et al, 2015(n=22)	1	8	0.1250	[0.0032; 0.5265]	0.9%	1.6%
Fanfani F et al, 2014(n=23)	0	6	0.0000	[0.0000; 0.4593]	0.6%	1.4%
Min CC et al, 2014(n=21)	3	10	0.3000	[0.0667; 0.6525]	1.1%	1.9%
Biliatis I et al, 2012(n=35)	0	7	0.0000	[0.0000; 0.4096]	0.8%	1.5%
Maneo A et al, 2011(n=36)	5	20	0.2500	[0.0866; 0.4910]	2.2%	2.6%
Fagotti A et al, 2011(n=17)	0	2	0.0000	[0.0000; 0.8419]	0.2%	0.6%
Baalbergen A et al, 2011(n=22)	4	18	0.2222	[0.0641; 0.4764]	1.9%	2.5%
Yahata T et al, 2010(n=10)	0	3	0.0000	[0.0000; 0.7076]	0.3%	0.8%
Lee SJ et al, 2009(n=85)	1	11	0.0909	[0.0023; 0.4128]	1.2%	2.0%
Maneo A et al, 2008(n=21)	1	10	0.1000	[0.0025; 0.4450]	1.1%	1.9%
Bisseling KCHM et al, 2007(n=18)	5	18	0.2778	[0.0969; 0.5348]	1.9%	2.5%
Landoni F et al, 2007(n=11)	0	3	0.0000	[0.0000; 0.7076]	0.3%	0.8%
Itsukaichi M et al, 2003(n=7)	0	2	0.0000	[0.0000; 0.8419]	0.2%	0.6%
Tseng CJ et al, 1997(n=12)	1	5	0.2000	[0.0051; 0.7164]	0.5%	1.2%
Morris M et al, 1993(n=14)	0	3	0.0000	[0.0000; 0.7076]	0.3%	0.8%
Fixed effect model		137	0.1475	[0.0934; 0.2116]	14.8%	--
Random effects model			0.1196	[0.0558; 0.2032]	--	24.6%
Heterogeneity: I-squared=39.8%, tau-squared=0.0199, p=0.0511						
**type = RT**						
Hauerberg L et al, 2015(n=120)	21	77	0.2727	[0.1774; 0.3862]	8.3%	3.6%
Vieira MA et al, 2015(n=100)	4	19	0.2105	[0.0605; 0.4557]	2.0%	2.5%
Jeong-Yeol P et al, 2014(n=55)	4	14	0.2857	[0.0839; 0.5810]	1.5%	2.2%
Lanowska M et al, 2014(n=20)	1	6	0.1667	[0.0042; 0.6412]	0.6%	1.4%
Faber-Swensson AP et al, 2014(n=17)	1	8	0.1250	[0.0032; 0.5265]	0.9%	1.6%
Ma LK et al, 2014(n=46)	5	15	0.3333	[0.1182; 0.6162]	1.6%	2.3%
van Gent MD et al, 2014(n=28)	0	14	0.0000	[0.0000; 0.2316]	1.5%	2.2%
Park JY et al,2014(n=79)	4	17	0.2353	[0.0681; 0.4990]	1.8%	2.4%
Kucukmetin A et al, 2014(n=16)	0	1	0.0000	[0.0000; 0.9750]	0.1%	0.3%
Capilna ME et al, 2014(n=26)	2	3	0.6667	[0.0943; 0.9916]	0.3%	0.8%
Lintner B et al, 2013(n=31)	1	4	0.2500	[0.0063; 0.8059]	0.4%	1.0%
Lu Q et al, 2013(n=25)	3	9	0.3333	[0.0749; 0.7007]	1.0%	1.8%
Ebisawa K et al, 2013(n=56)	5	31	0.1613	[0.0545; 0.3373]	3.3%	3.0%
Wethington SL et al, 2013(n=29)	7	21	0.3333	[0.1459; 0.5697]	2.3%	2.6%
Cao DY et al, 2013	1	1	1.0000	[0.0250; 1.0000]	0.1%	0.3%
Cao DY et al, 2013	9	23	0.3913	[0.1971; 0.6146]	2.5%	2.7%
Uzan C et al, 2013(n=28)	2	10	0.2000	[0.0252; 0.5561]	1.1%	1.9%
Li J et al, 2013(n=62)	0	3	0.0000	[0.0000; 0.7076]	0.3%	0.8%
Testa R et al, 2013(n=25)	0	2	0.0000	[0.0000; 0.8419]	0.2%	0.6%
Muraji M et al, 2012	0	1	0.0000	[0.0000; 0.9750]	0.1%	0.3%
Kim CH et al, 2012(n=105)	7	27	0.2593	[0.1111; 0.4628]	2.9%	2.8%
Persson J et al, 2012(n=12)	0	9	0.0000	[0.0000; 0.3363]	1.0%	1.8%
Persson J et al, 2012(n=13)	0	4	0.0000	[0.0000; 0.6024]	0.4%	1.0%
Raju SK et al, 2012(n=51)	2	16	0.1250	[0.0155; 0.3835]	1.7%	2.4%
Nick AM et al, 2012(n=37)	3	4	0.7500	[0.1941; 0.9937]	0.4%	1.0%
Wethington SL et al, 2012(n=101)	9	31	0.2903	[0.1422; 0.4804]	3.3%	3.0%
Saso S et al, 2012(n=30)	1	3	0.3333	[0.0084; 0.9057]	0.3%	0.8%
Plante M et al, 2011(n=125)	29	106	0.2736	[0.1915; 0.3687]	11.4%	3.7%
Li J et al, 2011(n=59)	0	2	0.0000	[0.0000; 0.8419]	0.2%	0.6%
Marchiole P et al, 2011(n=7)	0	1	0.0000	[0.0000; 0.9750]	0.1%	0.3%
Speiser D et al, 2011(n=212)	10	59	0.1695	[0.0844; 0.2897]	6.4%	3.4%
Yao T et al, 2010(n=10)	0	1	0.0000	[0.0000; 0.9750]	0.1%	0.3%
Kim JH et al, 2010(n=27)	2	3	0.6667	[0.0943; 0.9916]	0.3%	0.8%
Shepherd JH et al, 2009(n=142)	29	69	0.4203	[0.3024; 0.5452]	7.4%	3.5%
Olawaiye A et al, 2009(n=10)	2	4	0.5000	[0.0676; 0.9324]	0.4%	1.0%
Cibula D et al, 2009(n=24)	1	6	0.1667	[0.0042; 0.6412]	0.6%	1.4%
Nishio H et al, 2009(n=61)	0	3	0.0000	[0.0000; 0.7076]	0.3%	0.8%
Sonoda Y et al, 2008(n=40)	3	9	0.3333	[0.0749; 0.7007]	1.0%	1.8%
Milliken DA et al, 2008(n=158)	33	87	0.3793	[0.2774; 0.4897]	9.4%	3.6%
Pareja FR et al, 2008(n=15)	0	2	0.0000	[0.0000; 0.8419]	0.2%	0.6%
Shepherd JH et al, 2006(n=123)	23	54	0.4259	[0.2923; 0.5679]	5.8%	3.4%
Chen Y et al, 2006(n=16)	2	5	0.4000	[0.0527; 0.8534]	0.5%	1.2%
Ungar L et al, 2005(n=30)	1	3	0.3333	[0.0084; 0.9057]	0.3%	0.8%
Schlaerth JB et al, 2003(n=10)	2	4	0.5000	[0.0676; 0.9324]	0.4%	1.0%
Fixed effect model		791	0.2732	[0.2427; 0.3048]	85.2%	--
Random effects model			0.2395	[0.1875; 0.2957]	--	75.4%
Heterogeneity: I-squared=55.5%, tau-squared=0.0182, p<0.0001						
**Fixed effect model**		**928**	**0.2529**	**[0.2255; 0.2814]**	**100%**	**--**
**Random effects model**			**0.2056**	**[0.1612; 0.2539]**	**--**	**100%**
Heterogeneity: I-squared=55.6%, tau-squared=0.021, p<0.0001						

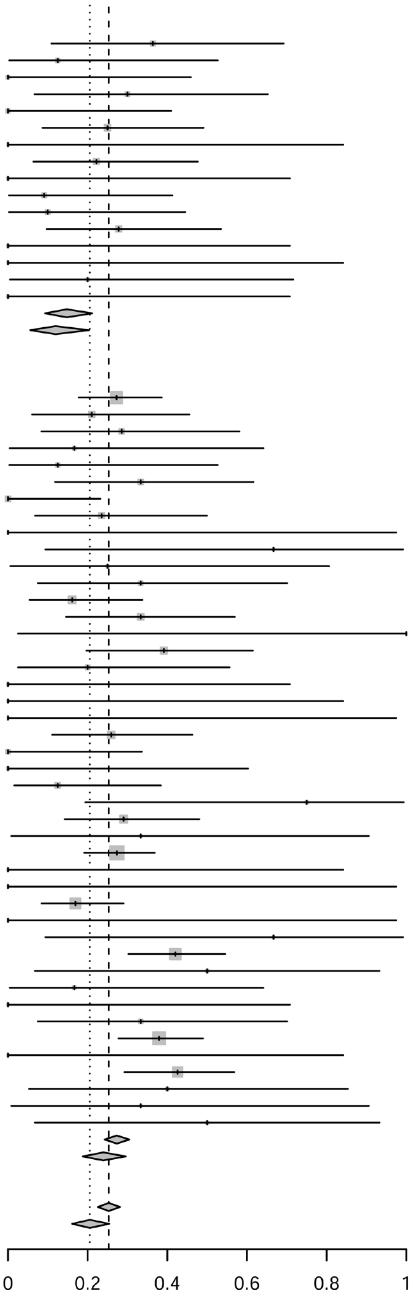

Supplementary Figure 3: Abortion rates of conization and RT.

Study	Events	Total	Proportion	95%-CI	W(fixed)	W(random)
type = CON						
Salihi R et al,2015(n=11)	2	11	0.1818	[0.0228; 0.5178]	1.2%	2.1%
Ditto A et al, 2015(n=22)	1	8	0.1250	[0.0032; 0.5265]	0.9%	1.9%
Fanfani F et al, 2014(n=23)	1	6	0.1667	[0.0042; 0.6412]	0.7%	1.7%
Min CC et al, 2014(n=21)	2	10	0.2000	[0.0252; 0.5561]	1.1%	2.0%
Biliatis I et al, 2012(n=35)	0	7	0.0000	[0.0000; 0.4096]	0.8%	1.8%
Maneo A et al, 2011(n=36)	3	20	0.1500	[0.0321; 0.3789]	2.3%	2.4%
Fagotti A et al, 2011(n=17)	0	2	0.0000	[0.0000; 0.8419]	0.2%	0.9%
Yahata T et al, 2010(n=10)	2	3	0.6667	[0.0943; 0.9916]	0.3%	1.2%
Lee SJ et al, 2009(n=85)	0	11	0.0000	[0.0000; 0.2849]	1.2%	2.1%
Landoni F et al, 2007(n=11)	0	3	0.0000	[0.0000; 0.7076]	0.3%	1.2%
Itsukaichi M et al, 2003(n=7)	0	2	0.0000	[0.0000; 0.8419]	0.2%	0.9%
Tseng CJ et al, 1997(n=12)	0	5	0.0000	[0.0000; 0.5218]	0.6%	1.5%
Morris M et al, 1993(n=14)	0	3	0.0000	[0.0000; 0.7076]	0.3%	1.2%
Fixed effect model		91	0.0778	[0.0320; 0.1413]	10.2%	--
Random effects model			0.0679	[0.0148; 0.1553]	--	20.9%
Heterogeneity: I-squared=40.3%, tau-squared=0.0251, p=0.0653						
type = RT						
Hauerberg L et al, 2015(n=120)	33	77	0.4286	[0.3163; 0.5465]	8.7%	2.9%
Vieira MA et al, 2015(n=100)	10	19	0.5263	[0.2886; 0.7555]	2.1%	2.4%
Jeong-Yeol P et al, 2014(n=55)	6	14	0.4286	[0.1766; 0.7114]	1.6%	2.3%
Lanowska M et al, 2014(n=20)	2	6	0.3333	[0.0433; 0.7772]	0.7%	1.7%
Faber-Swensson AP et al, 2014(n=17)	0	8	0.0000	[0.0000; 0.3694]	0.9%	1.9%
Ma LK et al, 2014(n=46)	4	15	0.2667	[0.0779; 0.5510]	1.7%	2.3%
van Gent MD et al, 2014(n=28)	0	14	0.0000	[0.0000; 0.2316]	1.6%	2.3%
Park JY et al,2014(n=79)	7	17	0.4118	[0.1844; 0.6708]	1.9%	2.4%
Kucukmetin A et al, 2014(n=16)	0	1	0.0000	[0.0000; 0.9750]	0.1%	0.5%
Capilna ME et al, 2014(n=26)	0	3	0.0000	[0.0000; 0.7076]	0.3%	1.2%
Lintner B et al, 2013(n=31)	1	4	0.2500	[0.0063; 0.8059]	0.5%	1.4%
Lu Q et al, 2013(n=25)	1	9	0.1111	[0.0028; 0.4825]	1.0%	2.0%
Ebisawa K et al, 2013(n=56)	17	31	0.5484	[0.3603; 0.7268]	3.5%	2.6%
Wethington SL et al, 2013(n=29)	10	21	0.4762	[0.2571; 0.7022]	2.4%	2.5%
Cao DY et al, 2013	0	1	0.0000	[0.0000; 0.9750]	0.1%	0.5%
Cao DY et al, 2013	4	23	0.1739	[0.0495; 0.3878]	2.6%	2.5%
Uzan C et al, 2013(n=28)	2	10	0.2000	[0.0252; 0.5561]	1.1%	2.0%
Li J et al, 2013(n=62)	0	3	0.0000	[0.0000; 0.7076]	0.3%	1.2%
Testa R et al, 2013(n=25)	2	3	0.6667	[0.0943; 0.9916]	0.3%	1.2%
Muraji M et al, 2012	1	1	1.0000	[0.0250; 1.0000]	0.1%	0.5%
Kim CH et al, 2012(n=105)	7	27	0.2593	[0.1111; 0.4628]	3.0%	2.6%
Persson J et al, 2012(n=12)	10	10	1.0000	[0.6915; 1.0000]	1.1%	2.0%
Persson J et al, 2012(n=13)	2	5	0.4000	[0.0527; 0.8534]	0.6%	1.5%
Raju SK et al, 2012(n=51)	0	16	0.0000	[0.0000; 0.2059]	1.8%	2.3%
Nick AM et al, 2012(n=37)	1	4	0.2500	[0.0063; 0.8059]	0.5%	1.4%
Wethington SL et al, 2012(n=101)	9	31	0.2903	[0.1422; 0.4804]	3.5%	2.6%
Saso S et al, 2012(n=30)	0	3	0.0000	[0.0000; 0.7076]	0.3%	1.2%
Plante M et al, 2011(n=125)	19	106	0.1792	[0.1115; 0.2657]	11.9%	2.9%
Li J et al, 2011(n=59)	0	2	0.0000	[0.0000; 0.8419]	0.2%	0.9%
Marchiole P et al, 2011(n=7)	0	1	0.0000	[0.0000; 0.9750]	0.1%	0.5%
Speiser D et al, 2011(n=212)	18	59	0.3051	[0.1919; 0.4387]	6.6%	2.8%
Yao T et al, 2010(n=10)	1	2	0.5000	[0.0126; 0.9874]	0.2%	0.9%
Kim JH et al, 2010(n=27)	1	3	0.3333	[0.0084; 0.9057]	0.3%	1.2%
Shepherd JH et al, 2009(n=142)	29	69	0.4203	[0.3024; 0.5452]	7.8%	2.8%
Olawaiye A et al, 2009(n=10)	1	4	0.2500	[0.0063; 0.8059]	0.5%	1.4%
Cibula D et al, 2009(n=24)	1	6	0.1667	[0.0042; 0.6412]	0.7%	1.7%
Nishio H et al, 2009(n=61)	2	4	0.5000	[0.0676; 0.9324]	0.5%	1.4%
Sonoda Y et al, 2008(n=40)	1	9	0.1111	[0.0028; 0.4825]	1.0%	2.0%
Milliken DA et al, 2008(n=158)	33	87	0.3793	[0.2774; 0.4897]	9.8%	2.9%
Pareja FR et al, 2008(n=15)	1	3	0.3333	[0.0084; 0.9057]	0.3%	1.2%
Shepherd JH et al, 2006(n=123)	28	54	0.5185	[0.3784; 0.6566]	6.1%	2.8%
Chen Y et al, 2006(n=16)	1	5	0.2000	[0.0051; 0.7164]	0.6%	1.5%
Ungar L et al, 2005(n=30)	0	3	0.0000	[0.0000; 0.7076]	0.3%	1.2%
Schlaerth JB et al, 2003(n=10)	1	4	0.2500	[0.0063; 0.8059]	0.5%	1.4%
Fixed effect model		797	0.3145	[0.2827; 0.3471]	89.8%	--
Random effects model			0.2660	[0.1961; 0.3423]	--	79.1%
Heterogeneity: I-squared=75.6%, tau-squared=0.0447, p<0.0001						
**Fixed effect model**		**888**	**0.2851**	**[0.2559; 0.3152]**	**100%**	**--**
**Random effects model**			**0.2134**	**[0.1537; 0.2801]**	**--**	**100%**
Heterogeneity: I-squared=75.5%, tau-squared=0.0512, p<0.0001						

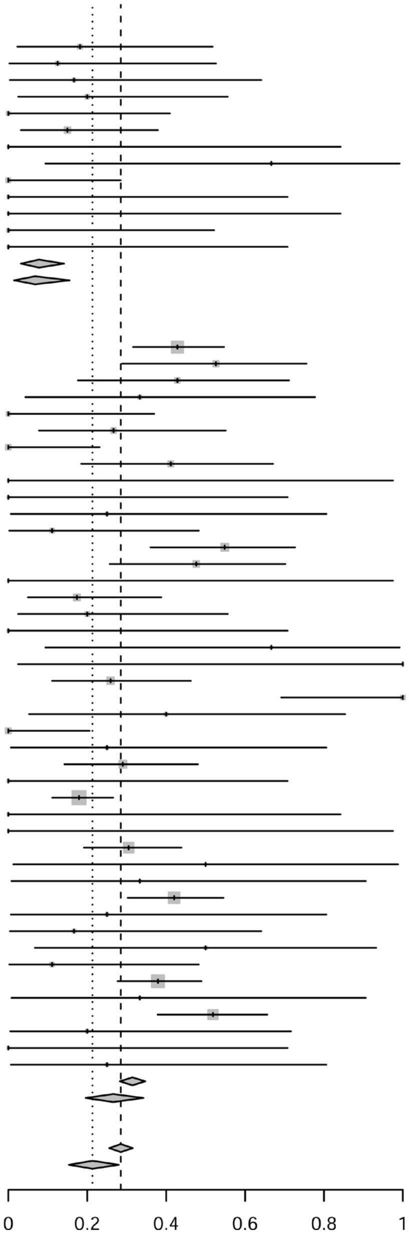

Supplementary Figure 4: Preterm delivery rates of conization and RT.
